# Evolution of Primary HIV Drug Resistance in a Subtype C Dominated Epidemic in Mozambique

**DOI:** 10.1371/journal.pone.0068213

**Published:** 2013-07-30

**Authors:** Dulce Celina Adolfo Bila, Peter Young, Harriet Merks, Adolfo Salvador Vubil, Mussagy Mahomed, Angelo Augusto, Celina Monteiro Abreu, Nédio Jonas Mabunda, James I. Brooks, Amilcar Tanuri, Ilesh Vinodrai Jani

**Affiliations:** 1 Instituto Nacional de Saúde, Maputo, Mozambique; 2 Laboratório de Virologia Molecular, Universidade Federal do Rio de Janeiro,Rio de Janeiro, Brazil; 3 Division of Global HIV/AIDS, Centers for Disease Control and Prevention (CDC), Maputo, Mozambique; 4 National HIV and Retrovirology Laboratories, National Microbiology Laboratory, Public Health Agency of Canada, Ottawa, Canada; 5 University of Ottawa, Ottawa, Canada; University of Pittsburgh, United States of America

## Abstract

**Objective:**

In Mozambique, highly active antiretroviral treatment (HAART) was introduced in 2004 followed by decentralization and expansion, resulting in a more than 20-fold increase in coverage by 2009. Implementation of HIV drug resistance threshold surveys (HIVDR-TS) is crucial in order to monitor the emergence of transmitted viral resistance, and to produce evidence-based recommendations to support antiretroviral (ARV) policy in Mozambique.

**Methods:**

World Health Organization (WHO) methodology was used to evaluate transmitted drug resistance (TDR) in newly diagnosed HIV-1 infected pregnant women attending ante-natal clinics in Maputo and Beira to non-nucleoside reverse transcriptase inhibitors (NNRTI), nucleoside reverse transcriptase inhibitors (NRTI) and protease inhibitors (PI). Subtypes were assigned using REGA HIV-1 subtyping tool and phylogenetic trees constructed using MEGA version 5.

**Results:**

Although mutations associated with resistance to all three drug were detected in these surveys, transmitted resistance was analyzed and classified as <5% in Maputo in both surveys for all three drug classes. Transmitted resistance to NNRTI in Beira in 2009 was classified between 5–15%, an increase from 2007 when no NNRTI mutations were found. All sequences clustered with subtype C.

**Conclusions:**

Our results show that the epidemic is dominated by subtype C, where the first-line option based on two NRTI and one NNRTI is still effective for treatment of HIV infection, but intermediate levels of TDR found in Beira reinforce the need for constant evaluation with continuing treatment expansion in Mozambique.

## Introduction

The sub-Saharan region of Africa is the most severely HIV affected region of the globe, housing more than two thirds (69%) of the people living with HIV in the world and 70% of the AIDS related deaths in 2011 [1]. Mozambique is one of nine countries in the region with an HIV prevalence above 10% and shares geographical boarders with Swaziland and South Africa, countries bearing the world’s highest adult HIV prevalence and the largest population of people living with HIV, respectively [1], [2] [1], [2]. Ante-natal clinic (ANC) HIV sentinel surveillance surveys from 2007 and 2009 indicate that the epidemic in Mozambique is stabilizing, with estimates of prevalence at 11.3% and 12.0%, respectively [2], [3]. A National Survey on Prevalence, Behavioral Risks, and Information about HIV and AIDS in Mozambique (INSIDA) in 2009 confirmed an adult HIV prevalence of around 11.5%, with highest rates in the southern region (17.8%), followed by the central region (12.5%) and northern region (5.6%) [2], [4].

Before 2003, only people that could afford treatment abroad had access to antiretroviral (ARV) treatment in Mozambique. Since 2004, efforts have been made to provide free and universal access to highly active antiretroviral therapy (HAART) with massive service expansion and integration of health services. As a consequence, the number of people on treatment has increased dramatically, reaching 170,198 people on treatment in 2009, a figure that represents 38% of people in need of HAART in Mozambique [5]. As directed by WHO guidelines, the most common first-line therapy used is based on a multi-drug approach which includes two NRTÍs and one NNRTI: usually Zidovudine (AZT), Lamivudine (3TC) and Nevirapine (NVP) or alternatively Stavudine (d4T), 3TC and NVP. Regimens for prevention of mother to child transmission of HIV (PMTCT) are also based on these drug classes. Patients failing first-line therapy are identified based on clinical and immunological criteria. Four options for second-line therapy are available based on the combination of Tenofovir, Lopinavir/Ritonavir (LPV/r); Abacavir, Didanosine and Saquinavir/Ritonavir [6], [7].

In resource-constrained countries such as Mozambique, ensuring viral suppression in patients on treatment to avoid drug resistance development and consequently the transmission of drug resistant strains is a priority. Despite efforts to build more robust systems for tracking and retention of patients, treatment default is still common. Monitoring of patients in care is suboptimal due to the lack of adequate infrastructure and expensive CD4 and viral load tests. Recently published results have indicated that loss to follow up (71%) was the main cause of 12-month attrition. The same study revealed that the rate of regimen switch to second-line options remains low, with only 0.56% of patients in care currently on second-line regimens [8].

These problems of treatment default, poor monitoring of patients in care, and delayed or absent switches to second-line therapies cause concern over the possibility of rapid development of HIV drug resistance (HIVDR). Samples collected in 2002 from 75 drug-naïve HIV-positive women from the southern, central and northern regions of Mozambique revealed no circulation of HIV drug resistance [9]. As HAART coverage expands exponentially and thousands of new people are placed on treatment every year, HIVDR and transmitted drug resistance (TDR) must be monitored.

WHO has developed a methodology for monitoring of TDR in resource-constrained countries that categorizes the level of resistance observed to each relevant drug class [10], [11]. Recent threshold surveys following this methodology in different African countries, including countries surrounding Mozambique such as Swaziland and South Africa, revealed that TDR rates remained below the WHO threshold limit of 5% [12]–[14].

In Mozambique, HIV drug resistance threshold surveys (HIVDR-TS) were conducted in Maputo (southern region) and Beira (central region), following WHO recommendations to conduct surveillance in capital cities where HAART has been available the longest and coverage is highest. Here we report the findings of two HIVDR-TS, conducted in 2007 and 2009 among pregnant women attending antenatal clinic (ANC).

## Materials and Methods

### Study area and Population

Two threshold surveys took place from March to June in 2007 and 2009 in Maputo (southern region) and Beira (central region), cities where antiretroviral programs were first introduced and expanded rapidly with HAART coverage in 2007 of 23% and 54% and in 2009 of 38% and 77% for Beira and Maputo respectively. Two health units from each city were included to ensure timely collection of samples within the surveillance round. Consistent with WHO guidance, specimens from both units within the same geographic area were pooled prior to assessment of TDR. (See Table 1).

**Table 1:** HAART service coverage at included antenatal care sites in Mozambique.

Geographic Area	HAART Coverage (%)	HAART Coverage (%)	Health Unit	Inclusion HIVDR-TS	Beginning of HAART Services
	2007	2009			
BEIRA	23	38	CS. Ponta Gea	2007; 2009	Jul, 2006
			CS. Chingussura	2007; 2009	May, 2003
	54	78	CS. José Macamo	2007; 2009	Jun, 2007
MAPUTO			CS. 1° de Junho	2009	Jan, 2007

The population for this survey consisted of pregnant women participating in the biannual ANC-based HIV surveillance program at five of the 36 sentinel sites in Mozambique. The lot quality assurance sampling (LQAS) methodology was used to classify TDR separately for each of the three drug classes [11]. To ensure collection of sufficient specimens to permit classification of TDR using this approach in each of the selected regions, a minimum of 47 women of those recruited for the surveillance program were sequentially selected for resistance testing based on the following inclusion criteria: age between 15–25 years old, first pregnancy, no prior exposure to ARVs, no prior HIV positive result, and HIV positivity confirmed by the National Reference Laboratory. In regions where 47 women could not be enrolled in a reasonable period of time the simplified LQAS (SLQAS) method was used. As opposed to the LQAS that categorizes TDR in three classes (low, intermediate and high), the SLQAS is a two class method where TDR is categorized in two classes (Low and High).

### Ethics Approval

ANC-based HIV surveillance is unlinked and anonymous. Specimens are prepared from blood specimens collected for routine RPR testing. Prior to initiation of surveillance procedures, oral consent was obtained from each participating subject and documented in the ANC register. Pregnant women aged 15–17 years were treated as emancipated minors and parental consent was not sought, following Mozambique health services guidelines for treating pregnant minors. All participants, regardless of consent to participate in surveillance, were offered PMTCT services. Protocols for both ANC surveys, including consent procedures, were approved by the National Ethics Review Committee in Mozambique and by the US Centers for Disease Control and Prevention.

### Specimen Collection and Processing

Five milliliters of blood were drawn from each woman for routine RPR screening. For women who consented to participate in ANC surveillance, dried blood spots (DBS) were prepared on Whatman 903 filter paper (Whatman, Inc, Sanford, USA). For each subject two DBS cards were prepared, dried overnight, and packed in a gas-impermeable zip-lock bag with five desiccant packs and one humidity indicator card, and stored in a refrigerator at 4–8°C before shipping to the reference laboratory in Mozambique. At the reference laboratory, two sequential ELISA tests were used to re-test the specimens. Specimens reactive to HIV antibodies on Vironostika HIV Uniform II plus O^1^ (Biomérieux, Durham, USA) were tested with Murex HIV 1–2–O^2^ (Abbott, UK) to confirm positivity, whereas non-reactive specimens were considered HIV-negative. Specimens eligible for HIVDR testing were stored in a freezer at-20°C, then shipped on dry ice to the National HIV and Retrovirology Laboratories of the Public Health Agency of Canada for genotyping**.**

### Sequencing and Analysis

Complete protease and part of reverse transcriptase were sequenced using the Big-Dye terminator Cycle Sequencing Kit (Applied Biosystems, California, USA) [15]. HIV subtype assignment based on the *pol* gene was performed using REGA and a phylogenetic tree constructed using neighbour-joining distance method [16], [17].

Sequences were assembled, edited, aligned to a reference subtype B sequence (accession number NC_001802), trimmed to identical length (1,241 bp) and had gaps removed using BioEdit Software, version 7.0.9. Analysis of drug resistance mutations was performed using the Stanford Drug Resistance Database (http: //hivdb.stanford.edu/hiv). For complete sequences subtyping was performed using the *REGA* HIV-1 subtyping tool version 2.0. Phylogenetic tree generation from complete PR (codons 1–99) and partial RT gene (codons 1–340) was conducted with *MEGA* 5 using Kimura’s two parameter model (2 K-P) neighbour-joining analysis with 1000 bootstraps [16]–[18].

### Classification of Transmitted HIV Drug Resistance

In addition to reporting the type and frequency of drug mutations encountered, the LQAS method recommended by WHO for HIVDR surveillance was also applied for areas where the minimum required number of specimens were obtained and the SLQAS for areas where the minimum numbers could not be obtained due to low incidence or time constraints [19]. Both LQAS and SLQAS algorithms were applied separately for each drug class (NNRTI, NRTI and PI), for each geographic area (Maputo, Beira), and for each survey (2007, 2009). In the case of LQAS, HIVDR was classified as low (<5%), medium (5–15%), or high (>15%). Briefly, for regions where 47 specimens were collected, sequences from the first 34 specimens collected were analyzed and the number of primary mutations from the WHO surveillance list for the drug class in question was counted. If no mutations were encountered, resistance was classified as <5%. If at least one mutation was encountered an additional 13 sequences were analyzed and if only one mutation was encountered resistance was classified as <5%. If the first 47 sequences had 6 or more listed mutations, resistance was classified as >15%, otherwise it was classified as 5–15%. If fewer than the minimum number of sequences was available for LQAS, a SLQAS algorithm was used requiring a single cutoff which was set to detect TDR above or below 10%, i.e. the average of the low and high cutoffs used for three-category LQAS. The exact decision rule was calculated based on the achieved sample size [19]. The algorithms were repeated for each combination of drug class, geographic area, and survey year.

## Results

Due to quality problems in specimen collection one site from Maputo was excluded in 2007. The mean age of the participants was 20.3 years in 2007 and 20.9 in 2009. Summing across regions, 102 and 132 specimens were collected from 2007 and 2009 surveys, respectively. Serological confirmation followed by genotyping yielded 75 (73.5%) reportable sequences in 2007 and 114 (84.8%) reportable sequences in 2009 for the PR region and 64 (62.8%) reportable sequences in 2007 and 123 (93.2%) reportable sequences in 2009 for the RT region, reflecting an improvement in DBS quality and storage conditions in 2009 when compared to 2007.

The SLQAS algorithm was used for analysis of TDR in Maputo for the 2007 survey. Maputo had 21 specimens available for classification of TDR for NRTI and NNRTI, and only 23 specimens were available for TDR classification for PI. Based on the 10% cutoff for the SLQAS algorithm, prevalence of TDR for all three drug classes was found to be <10%. Nonetheless one sequence carrying a PI mutation (M46L) was identified. Sequential analysis of the first 34 specimens from the same survey year from Beira for PI and NNRTI mutations did not show the occurrence of mutations, thus a prevalence of <5% for both PI and NNRTI drug classes was assigned. In contrast we found 2 NRTI mutations (M41L) conferring resistance to thymidine analogs in the first 34 specimens analyzed. Subsequential evaluation of four additional specimens revealed no drug resistance mutations, thus TDR was classified as intermediate to NRTI (see Table 2).

**Table 2:** Transmitted drug resistance (TDR) classification and subtype assignment from two threshold surveys conducted in Mozambique in 2007 and 2009.

Year	Region	Eligible	Reportable	Classification	N° of sequences	TDR Classification	Subtype	Discordant
		Samples (a)	sequences	(N° evaluated)	with Mutations	(Algorithm)	Assignment (b)	Subtype
			per region		(Mutations)			assignment (c)
			PR (43)	PI (34)	0	<5% (LQAS)		
	BEIRA	77	RT (52)	NRTI (47)	2 (M41L)	5–15% (LQAS)	C	0
2007				NNRTI (34)	0	<5% (LQAS)		
			PR (23)	PI (23)	1 (M46L)	<10% (SLQAS)		
	MAPUTO	25	RT (21)	NRTI (21)	0	<10% (SLQAS)	C	0
				NNRTI (21)	0	<10% (SLQAS)		
			PR (70)	PI (34)	0	<5% (LQAS)		
	BEIRA	76	RT (67)	NRTI (34)	0	<5% (LQAS)	C	1 (H/C)
					1 (K101E)			
2009				NNRTI (47)	2 (K103N)	5–15% (LQAS)		1 (D/C)
			PR (53)	PI (34)	0	<5% (LQAS)		
	MAPUTO	56	RT (47)	NRTI (34)	0	<5% (LQAS)	C	0
				NNRTI (34)	0	<5% (LQAS)		

(a) A total of 77+25 = 102 samples were eligible in 2007 and 76+56 = 132 samples were eligible in 2009.

(b) Subtype assigned by REGA HIV-1 subtyping tool version 2.0 and confirmed by phylogenetic analysis.

(c) Discordant results as per subtype assigned by Stanford Drug Resistance Database.

In 2009 the minimum sample size required for use with LQAS was attained for both regions. Consecutive analysis of 34 specimens from Maputo showed the absence of PI, NRTI and NNRTI relevant mutations, thus the prevalence was categorized as <5% for all drug classes. On the other hand analysis of the first 34 samples collected in Beira showed the presence of a NNRTI mutation at position K101E (n = 1) and K103N (n = 2) (Table 2). Since the upper limit for the number of sequences with relevant resistance mutations was reached we analyzed an additional thirteen specimens to better categorize the TDR. No additional drug resistance mutations were found, thus TDR prevalence was categorized as between 5–15% for NNRTIs in 2009 in Beira.

**Figure 1:**
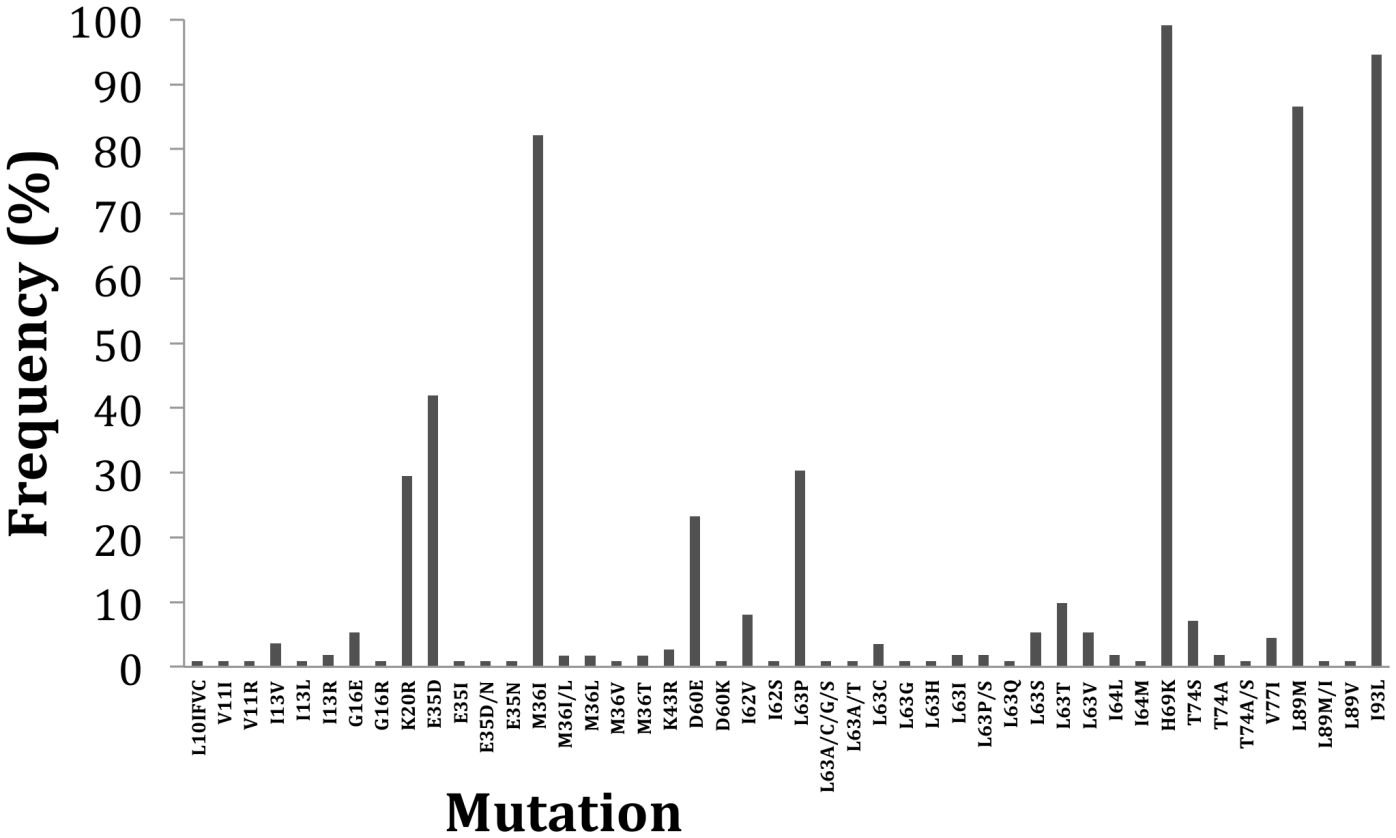
Distribution of PI secondary mutations in 112 specimens collected in Maputo and Beira in 2009.

**Figure 2:**
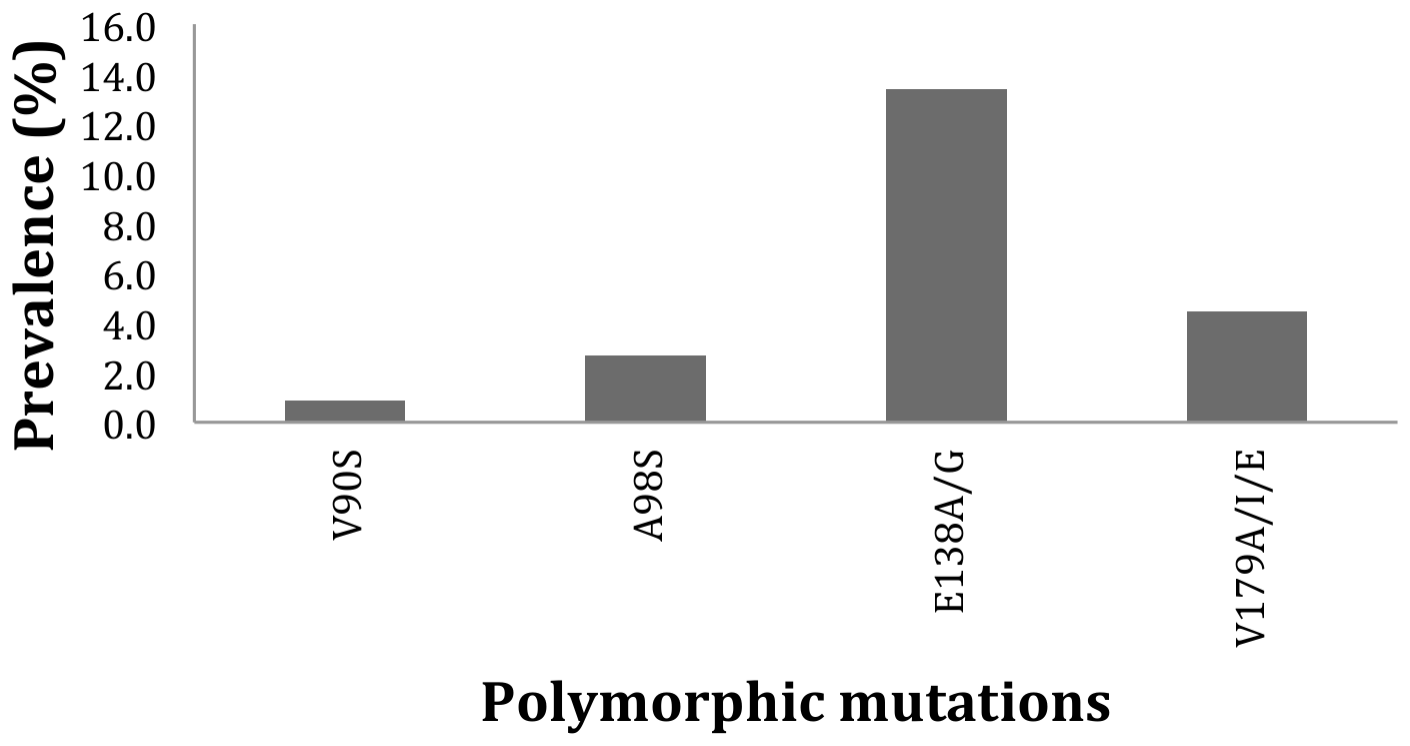
Distribution of NNRTI (etravirine) secondary mutations in 112 specimens collected in Maputo and Beira in 2009.

In total, 112 specimens from the 2009 survey were analyzed for both geographic areas and the majority of PI mutations found could be classified as minor mutations with the exception of Q58E which can be a nonpolymorphic PI-selected mutation associated with decreased susceptibility to TPV/r (Figure 1). Similarly, secondary mutations to etravirine were identified in these sequences [E138A/G (13.4%) and V179A/I/E (4.9%)]. Unusual substitutions at secondary mutation position V90I and A98G were identified V90S and A98S, respectively, suggesting the occurrence of polymorphisms at these positions (Figure 2). Likewise, intermediate mutation at known resistant site K103 and V179 were identified: K103R (n = 1, 0.9%), V179A (n = 1, 0.9%), V179E (n = 1, 0.9%) and V179I (n = 3, 2.7%) (Data not shown).

**Figure 3:**
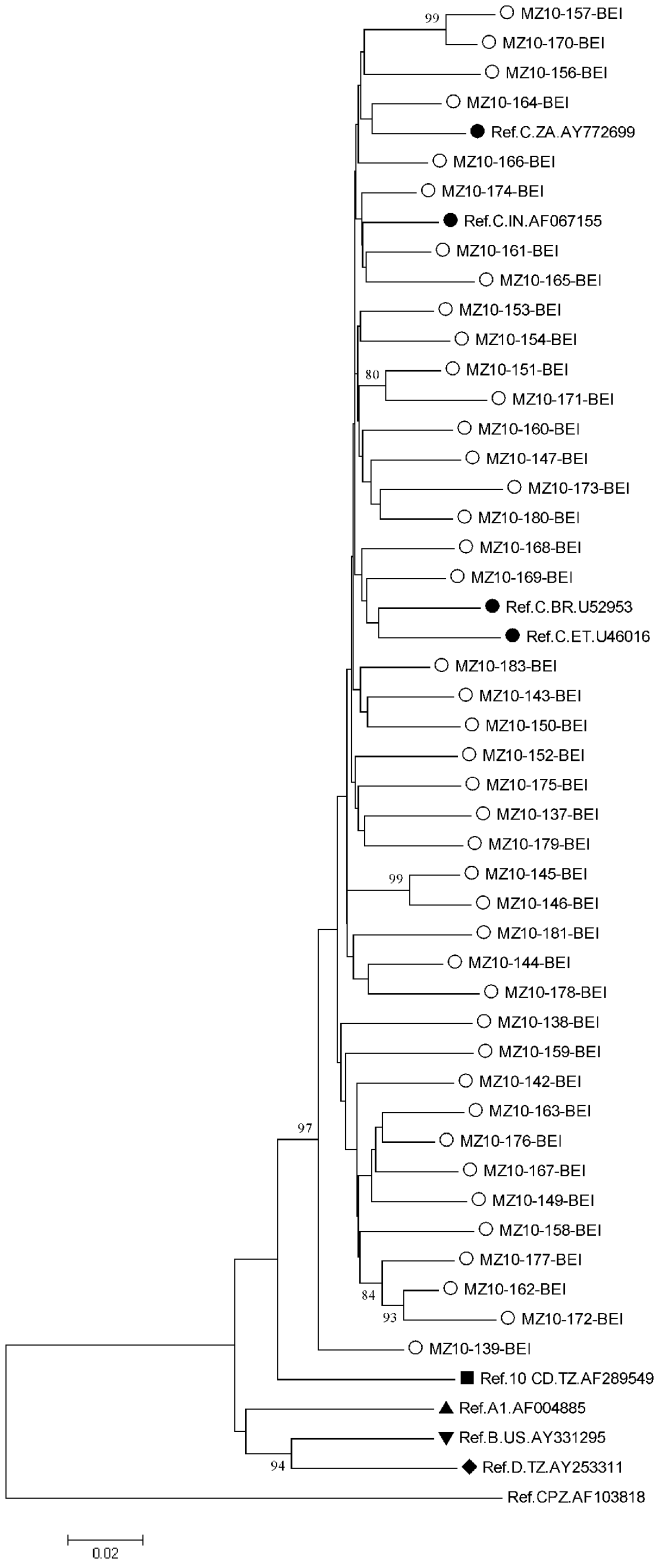
Phylogenetic tree depicting HIV sequences from Beira analyzed in 2009 TDR threshold survey. Tree was constructed bases on complete sequences of protease and part of RT gene (1.317 bp), using Kimura 2-parameter (K2P) model of base substitution with bootstrap analysis (100 replications) in MEGA 5.05. Nomenclature of samples herein characterized is as follows: MZ (country) and year of sequencing-patient code-region of isolation [BEI: Ponta Gea Health Center and Chingussura Health Center]. Tree includes sequences of the HIV-1 subtypes inferred through REGA (empty circles) along with previously described subtype C in the region, India and Brazil (full circles), other subtypes (other full shapes) and outlier group CPZ, obtained from Los Alamos HIV Sequence Database Subtype Reference Alignments (http: //www.hiv.lanl.gov/content/index).

**Figure 4:**
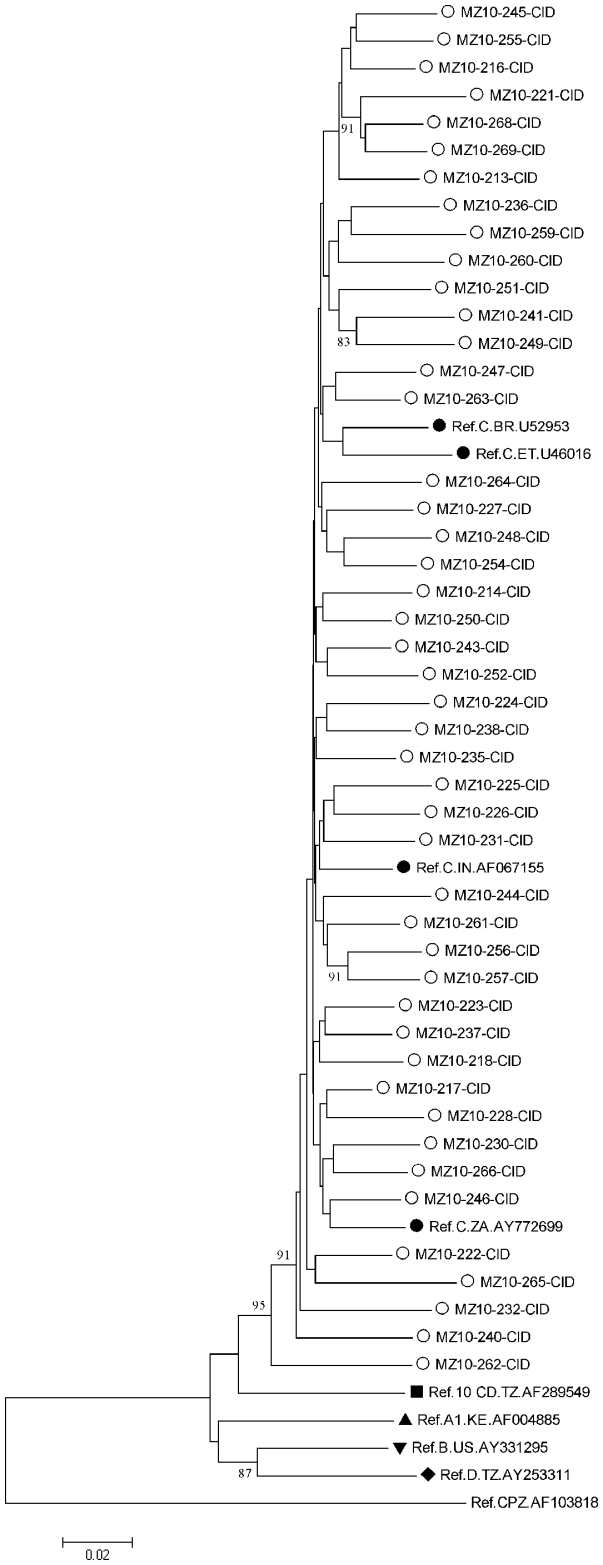
Phylogenetic tree depicting HIV sequences from Maputo analyzed in 2009 TDR threshold survey. Tree was constructed bases on complete sequences of protease and part of RT gene (1.317 bp), using Kimura 2-parameter (K2P) model of base substitution with bootstrap analysis (100 replications) in MEGA 5.05. Nomenclature of samples herein characterized is as follows: MZ (country) and year of sequencing-patient code-region of isolation [CID: Jose Macamo Health Center and 1° de Junho Health Center]. Tree includes sequences of the HIV-1 subtypes inferred through REGA (empty circles) along with previously described subtype C in the region, India and Brazil (full circles), other subtypes (other full shapes) and outlier group CPZ, obtained from Los Alamos HIV Sequence Database Subtype Reference Alignments (http: //www.hiv.lanl.gov/content/index).

In order to confirm the subtype assignment done by *REGA*, phylogenetic analysis of 112 sequences representing the 2009 survey in Maputo and Beira was performed and results are depicted in Figure 3 and 4; all clustered phylogenetically with HIV-1 subtype C (Genebank access numbers JX507809 to JX507920).

## Discussion

In this study we report for the first time the occurrence of TDR in Mozambique using the WHO-recommended methodology for TDR surveillance among recently infected pregnant women. In a context of rapid scaling-up and decentralization of HAART services data generated from such studies are of extreme relevance to the country as these may be used by decision makers for choice of therapeutic options, improvement of prevention and treatment programs and treatment monitoring strategies. Analysis was performed based on the mutations list from WHO [20]. Due to difficulties with specimen collection and storage it was not possible to evaluate the minimum number of specimens required for the LQAS method in every region for both years. However, the use of the SLQAS methodology for specimens from Maputo, allowed the classification of TDR in this region in 2007 in spite of the reduced sample size. This analysis showed low prevalence of TDR for all drug classes but the identification of PI mutation M46L raises concerns regarding the influence of this transmitted drug resistant mutation on second-line PI-based treatment regimens and underlines the potential value of genotyping prior to second-line initiation. Interestingly, only 0.56% of patient on HAART in Mozambique are on second-line treatment [7], thus the occurrence of such mutations may suggest the possible impact of treatment programs from neighbouring countries where PIs are more widely used.

Of note, NRTI and NNRTI mutations were not identified in Maputo in contrast to Beira where NRTI mutation M41L was found in two sequences. This mutation confers resistance to stavudine (d4T) and zidovudine (AZT), antiretrovirals that compose the first-line backbone in Mozambique [6], [7]. In 2009, TDR in Maputo was low and was classified as <5% for each of the three drug classes analyzed (NRTI, NNRTI and PI). On the other hand, two NNRTI mutations (K101E, n = 1) and (K103N, n = 2) were found in Beira. Mutation K101E confers intermediate resistance to NVP and delavirdine (DLV) and low-level resistance to EFV and etravirine (ETR), whereas mutation K103N causes high-level resistance to NVP, DLV, and EFV. These mutation patterns fit the ARV profile used for first-line treatment options as well as for PMTCT. Not unexpectedly, high prevalence of secondary PI mutations was found as these may appear as natural polymorphisms in non-B subtypes found in-country. Interestingly, the patterns of polymorphisms found in the present study differed in comparison to early results obtained from a European cohort for subtype-B. In that study the most prevalent polymorphisms in subtype B isolates were L63P (44.2%), V77I (18.9%), M36I (17.2%) and L10I (5.7%) [21]. In subtype C circulating in Mozambique, we found signature substitutions to be H69K (99.1%), I93L (94.6%), L89M (86.6%), M36I (82.1%), K20R (29.46%), D60E (23.21%), I62V (8.03%) and G16E (5.35%). Some of the secondary mutations found in our study confirm the most common secondary mutations found in 2003 in Maputo by Bellocchi et al. [22]. Polymorphisms at primary resistance site T74S (7.1%) and T74S/A (0.89%) were also found. T74S is a common polymorphism found in subtype C viruses circulating in Africa and South America, and it is associated with reduced nelfinavir susceptibility. All together these results give support to the suggestion that different virus subtypes may show different drug mutational pathways and the impact of this pattern on natural resistance to PI or unusual susceptibility is unknown [21]–[24].

The prevalence of TDR found in Maputo is similar to prevalence found in threshold surveys performed in surrounding countries such as Swaziland, South Africa, Malawi and Tanzania [14], [23], [25], [26]. Mutations found in our study were in accordance with the therapeutic lines in use in Mozambique, as well as drugs used for PMTCT. Results from previous studies suggest that the epidemic has been dominated by subtype C in Mozambique [9], [27]. Our results are consistent with these findings confirming reports from different sub-Saharan countries that suggest that subtype C is the major subtype circulating in this part of the globe. An early study done in Mozambique published in 2008 by Abreu et al, with subjects with the same characteristics did not find drug resistance mutations, a result that can be justified by the fact that treatment introduction and subsequent expansion started in late 2004. Nevertheless the same study found that other subtypes and HIV mosaics circulate in Mozambique.

Sample collection in ANC settings for HIVDR-TS presents challenges and difficulties with sample collection limited our ability to fully apply the LQAS algorithm in all regions for all drug classes in both surveys. However, application of the simplified LQAS algorithm allowed us to make full use of available specimens. Two consecutive surveys, in 2007 and 2009, have detected intermediate levels of TDR in Beira, thus revealing the need for intensification of prevention programs, along with the implementation of continued monitoring of drug resistance in other regions of the country. Moderate classification of TDR needs to be confirmed through repetition of the survey in this area and possibly in additional areas of the country. Furthermore work should be done to identify possible sources of HIVDR transmission, through evaluation of prevention programs and intensification of HIVDR early warning indicators monitoring.
